# Time-to-Positivity of Blood Cultures in Children With Sepsis

**DOI:** 10.3389/fped.2018.00222

**Published:** 2018-08-08

**Authors:** Alexa Dierig, Christoph Berger, Philipp K. A. Agyeman, Sara Bernhard-Stirnemann, Eric Giannoni, Martin Stocker, Klara M. Posfay-Barbe, Anita Niederer-Loher, Christian R. Kahlert, Alex Donas, Paul Hasters, Christa Relly, Thomas Riedel, Christoph Aebi, Luregn J. Schlapbach, Ulrich Heininger, Swiss Pediatric Sepsis Study

**Affiliations:** ^1^Infectious Diseases and Vaccinology, University of Basel Children's Hospital, Basel, Switzerland; ^2^Division of Infectious Diseases and Hospital Epidemiology, Children's Research Center, University Children's Hospital Zurich, Zurich, Switzerland; ^3^Department of Pediatrics, Inselspital, Bern University Hospital, University of Bern, Bern, Switzerland; ^4^Cantonal Hospital Aarau, Children's Hospital, Aarau, Switzerland; ^5^Department Mother-Woman-Child, Service of Neonatology, Lausanne University Hospital, Lausanne, Switzerland; ^6^Infectious Diseases Service, Lausanne University Hospital, Lausanne, Switzerland; ^7^Department of Pediatrics, Children's Hospital Lucerne, Lucerne, Switzerland; ^8^Pediatric Infectious Diseases Unit, Children's Hospital of Geneva, University Hospitals of Geneva, Geneva, Switzerland; ^9^Children's Hospital of Eastern Switzerland St. Gallen, St. Gallen, Switzerland; ^10^Department of Neonatology, University Hospital Zurich, Zurich, Switzerland; ^11^Department of Pediatrics, Cantonal Hospital Graubuenden, Chur, Switzerland; ^12^Faculty of Medicine, The University of Queensland, Brisbane, QLD, Australia; ^13^Paediatric Critical Care Research Group, Mater Research Institute, University of Queensland, Brisbane, QLD, Australia; ^14^Paediatric Intensive Care Unit, Lady Cilento Children's Hospital, Children's Health Queensland, Brisbane, QLD, Australia

**Keywords:** sepsis, children, bacteremia, blood cultures, time-to-positivity

## Abstract

**Background:** Blood cultures are essential for the diagnosis and further appropriate treatment in children with suspected sepsis. In most hospitals, children will be empirically treated or closely monitored for at least 48 h awaiting results of blood cultures. Several studies have challenged the optimal duration of empiric treatment in the era of continuously monitored blood culture systems. The aim of our study was to investigate time-to-positivity (TTP) of blood cultures in children with proven sepsis.

**Methods:** The Swiss Pediatric Sepsis Study prospectively enrolled children 0–16 years of age with blood culture positive sepsis between September 2011 and October 2015. TTP was prospectively assessed in six participating academic pediatric hospitals by fully automated blood culture systems.

**Results:** In 521 (93%) of 562 bacteremia episodes (493 children, median age 103 days, range 0 days−16.9 years) a valid TTP was available. Median TTP was 12 h (IQR 8–17 h, range 0–109 h). By 24, 36, and 48 h, 460 (88%), 498 (96%), and 510 (98%) blood cultures, respectively, were positive. TTP was independent of age, sex, presence of comorbidities, site of infection and severity of infection. Median TTP in all age groups combined was shortest for group B streptococcus (8.7 h) and longest for coagulase-negative staphylococci (16.2 h).

**Conclusion:** Growth of bacteria in blood cultures is detectable within 24 h in 9 of 10 children with blood culture-proven sepsis. Therefore, a strict rule to observe or treat all children with suspected sepsis for at least 48 h is not justified.

## Introduction

Surviving Sepsis Campaign guidelines recommend obtaining blood cultures before initiation of antibiotic treatment in newborns, infants and children with suspected sepsis (1, 2). Due to globally increasing antibiotic resistance rates, the World Health Organization (WHO) has urged countries to develop action plans against antibiotic resistance (3). Blood cultures represent a cornerstone of antibiotic stewardship to streamline targeted treatment and to reduce unnecessary use of antibiotics. However, blood cultures in patients with suspected sepsis are often negative. For example, the number of neonates with suspected sepsis needed to treat for 1 culture proven neonatal sepsis varies between 44 and 100 (4–6).

In most hospitals, children with suspected sepsis will be empirically treated or closely monitored for at least 48 h awaiting results of the blood cultures. However, this measure is based on limited evidence. The American Academy of Pediatrics recommends stopping antibiotic therapy in neonates treated for suspected early-onset sepsis after 48 h if cultures remain negative (7). Several studies in infants <90 days of age have challenged whether this empiric treatment and observation period is still justified in the era of continuous monitoring blood culture systems (8–10): They demonstrated that ≥96% of all blood cultures were positive by 36 h (9, 10) and that ceasing antibiotic treatment after implementation of an evidence-based care process model including an empiric treatment period of 24–36 h did not show any adverse events (8).

Limitations of currently available data on optimal use of time-to-positivity (TTP) for decision making on empiric antibiotic treatment in suspected sepsis are (1) lack of data on pediatric age groups beyond early infancy, (2) lack of multicenter studies, and (3) lack of detailed characterization of host and pathogen features. The aim of this study was to analyse TTP in children with blood culture positive sepsis recruited through a large prospective multicenter population-based pediatric sepsis cohort study and to investigate host and pathogen factors associated with TTP.

## Methods

As part of the Swiss Pediatric Sepsis Study, we analyzed TTP values in children with blood culture positive sepsis between September 2011 and October 2015. Details of the Swiss Pediatric Sepsis Study have previously been reported (11, 12). The study was approved by the respective ethics committees of all participating centers (Cantonal Ethics Committee, Inselspital, University of Bern, no. KEK-029/11). Written informed consent from patients, and/or their legal guardians was obtained before study enrollment. In patients who fulfilled inclusion criteria but consent was not available (224 of 521), waiver from informed consent had been granted by the ethics commission for collection of anonymized clinical data.

Children 0–16 years of age with bacteremia in the presence of a systemic inflammatory response syndrome (SIRS) were enrolled in all 10 major pediatric hospitals in Switzerland during the study period. SIRS, severe sepsis and septic shock were defined according to the international consensus conference on pediatric sepsis (13). Briefly, for children beyond the neonatal period, SIRS was defined as the presence of at least 2 of the following four criteria, one of which had to be abnormal temperature or leukocyte count: body temperature <36°C or >38.5°C; abnormal heart rate for age; abnormal respiratory rate for age; leukocyte count elevated or depressed for age or >10% immature neutrophils. Age specific limits for heart rate, respiratory rate, and leukocyte count were applied as defined in the 2005 consensus definitions. For neonates (<28 days old, or <44 weeks postconceptional age in premature newborns) at least 2 of the following signs were required to be present: tachycardia >180/min, tachypnea >60/min or increased apnea frequency, temperature instability, leukocyte count 34 × 103 /mm^3^ or immature:total neutrophil ratio >0.2, capillary refill >2 s, apathia or irritability.

Severe sepsis was defined as sepsis plus one of the following: cardiovascular organ dysfunction or acute respiratory distress syndrome or two or more other organ dysfunctions.

Septic shock was defined as sepsis and cardiovascular organ dysfunction.

Each bacteremia episode received a study number and was analyzed. The present sub-study is restricted to 6 of the 10 participating centers (designated A to F) where fully automated blood culture systems were used, which allowed prospective assessment of TTP. TTP was defined as the time interval between placement of the blood culture bottle into the automated system and detection of a positive signal. The following blood culture systems were used: Beckton Dickinson (BD), Allschwill, Switzerland, Bactec Aerob/Anaerob, Peds Plus/F und mycosis IC/F Mycosis und Pedi (center A), BD Bactec FX (center B), BD Bactec Lytic/10 Anaerobic/F, BD Bactec Aerobic Plus/F BD Bactec Peds Plus/F (center C), bioMerieux, Geneva, Switzerland, BacT/ALELRT PF Aerob/anaerob, BacT/ALERT FA Aerob, BacTALERT FN Anaerob (center D), BD Bactec peds plus/F, BD Bactec lytic/10 anaerobic/f, BD Bactec plus aerobic/F (center E), BD Bactec plus aerobic/F, BD Bactec plus anaerobic/F, BD Bactec peds plus (center F). Centers B to F used fully automated blood culture systems from the beginning of the study, center A introduced it mid-2013.

Early-onset neonatal sepsis was defined as sepsis occurring <72 h of age and late-onset neonatal sepsis as ≥72 h in term infants and 72 h of age to <44 weeks of gestational age in premature infants. Hospital-acquired infection (i.e., blood culture obtained >48 h after admission) and central line-associated bloodstream infection (CLABSI) were defined according to the criteria of the Centers for Disease Control and Prevention (CDC) (14). In patients with suspected sepsis, positive blood cultures were only included if contamination was ruled out by the treating physician. The definition of contamination was based on the following criteria: absence of a central line at the time the blood culture was taken; blood culture growing a mixed flora of different coagulase-negative staphylococci (CoNS); and blood cultures growing pathogens considered as contaminants by the treating physician.

Chronic inborn or acquired medical conditions, recent surgery, or burns where considered as comorbidities and were categorized according to the pediatric complex chronic conditions classification system, version 2 (15).

### Pre-planned subgroup analyses and statistics

For statistical data analysis all extracted data was stored in spreadsheets (Microsoft Excel 2010, version 14.0). For descriptive analyses, the total number of cases and all cases stratified by study center were analyzed. For comparative analyses of TTP, we analyzed cases from all study centers combined and all bacteria combined, stratified by sex. For further analyses, cases were stratified by study centers, the 5 most frequently isolated organisms, age (0–27 days, 28–365 days, 1–5 years, 6–10 years and >10 years), focus of infection, outcome/severity of infection, prematurity, comorbidity, and community-acquired vs. nosocomial infection.

Descriptive statistics are presented as median (IQR) for continuous variables and as frequencies (%) for categorical variables. We fitted a generalized linear model with a log-link function and Gamma distribution for potential determinants of TTP with age, gender, presence of comorbidity, sepsis severity, site of infection, and pathogens using a random effect to correct for correlation between multiple observations at the same hospital. We added 0.01 h to all TTP measurements to remove zero values. We separately fitted univariable models or all variables and a multivariable model containing all variables. We present the results of the multivariable model as estimates (multiplicative factor by which to multiply the TTP of the respective reference group) with 95% CIs and *p*-values of likelihood ratio testing. We additionally fitted a multilevel binomial regression model for potential determinants of death within the first 30 days after sepsis onset with the TTP (adjusting for patient age, sex, presence of comorbidity, severity of sepsis, site of infection, and pathogen) using a random effect to correct for correlation between multiple observations at the same hospital.

Statistical significance was defined at a two-sided *p*-value of <0.05. We did regression analyses with R version 3.4.3.

## Results

### General characteristics

During the study period, 493 patients with 562 bacteremia episodes were enrolled in study centers A-F. Of these, 521 (93%) had blood culture TTP recorded and they are the subject of these analyses; 311 (60%) of 521 episodes occurred in male patients with no difference between infants <90 days of age and patients >90 days of age (Supplement Table 1). Median age of patients was 103 days (range: 0 days−16.9 years; IQR 11 days−3.8 years); 196 (38%) were neonates (i.e., ≤28 days old), 257 (49%) were <90 days old and 125 (24%) were <365 days old.

In 291 (56%) episodes the sepsis was community-acquired and 86 (30%) of these patients had at least 1 comorbidity. In 230 (44%) episodes the sepsis was hospital-acquired and 205 (89%) of these patients had at least 1 comorbidity.

In 417 (80%) episodes a focus for sepsis could be identified and most commonly this was related to a central venous catheter Case fatality rate was 7% (*N* = 37). Children <90 days of age had significantly more episodes with severe sepsis than children >90 days of age (Supplement Table 1).

Blood culture isolates by study center are summarized in Table 1. Overall 336 (64%) were Gram-positive and 185 (36%) were Gram-negative pathogens. The distribution of pathogens stratified by age, i.e., <90 days and >90 days, is shown in Supplement Table 1.

**Table 1:** Bacterial pathogens isolated from blood cultures by study center (A-F).

	**A n (%)**	**B n (%)**	**C n (%)**	**D n (%)**	**E n (%)**	**F n (%)**	**Total n (%)**
*Escherichia coli*	21 (20)	6 (11)	18 (13)	21 (24)	23 (24)	8 (22)	97 (19)
Coagulase-negative-staphylococci	7 (7)	15 (28)	40 (29)	6 (7)	13 (13)	2 (5)	83 (16)
*Staphylococcus aureus*	13 (12)	9 (17)	18 (1)	11 (12)	13 (13)	8 (22)	77 (14)
Group B streptococcus	11 (10)	12 (22)	4 (3)	4 (4)	10 (10)	3 (8)	47 (9)
*Streptococcus pneumoniae*	13 (12)	1 (2)	9 (6)	8 (9)	8 (8)	8 (22)	47 (9)
Group A streptococcus	5 (5)	0 (0)	4 (3)	8 (9)	5 (5)	2 (5)	24 (5)
*Enterococcus* spp.	8 (8)	1 (2)	4 (3)	2 (2)	2 (2)	2 (5)	19 (4)
*Klebsiella* spp.	7 (7)	1 (2)	11 (8)	1 (1)	4 (4)	1 (3)	25 (5)
*Neisseria meningitidis*	2 (2)	0 (0)	4 (3)	3 (3)	4 (4)	2 (5)	13 (2)
*Pseudomonas aeruginosa*	4 (4)	0 (0)	3 (2)	1 (1)	2 (2)	0 (0)	11 (2)
*Haemophilus influenzae*	1 (1)	0 (0)	2 (1)	1 (1)	3 (3)	0 (0)	6 (1)
Other Gram-negative pathogens^♦^	11 (10)	2 (4)	10 (7)	2 (2)	4 (4)	0 (0)	29 (6)
Other Gram-positive pathogens^•^	2 (2)	7 (13)	15 (11)	6 (7)	6 (6)	1 (3)	38 (7)
Total	105	54	139	89	97	37	521

♦ Stenotrophomonas maltophilia, Serratia marcescens, Serratia liquefaciens, Citrobacter Sedlakii, Citrobacter freundii, Elizabethkingia meningoseptica, Salmonella group B, Salmonella group C, Acinetobacter Iwofii, Enterobacter cloacae, Capnocytophaga sp., Proteus mirabilis, Cardiobacterium hominis

• *Bifidobacterium spp., Fusobacterium nucleatum, Fusobacterium necrophorum, Streptococcus bovis, Lactobacillus spp., Streptococcus mitis, Streptococcus equi, Streptococcus, salivarius, Streptococcus anginosus, Streptococcus sanguinis, Streptococcus viridans, Listeria monocytogenes, Micrococcus luteus, Bacillus cereus, Bacillus pumilus, Rothia mucilaginosa*,

### Time to positivity and associations with pathogens and host characteristics

Median TTP was 11.7 h (IQR 8.3–17.1) with minimal variability between different study centers (Supplement Table 2).

**Figure 1:**
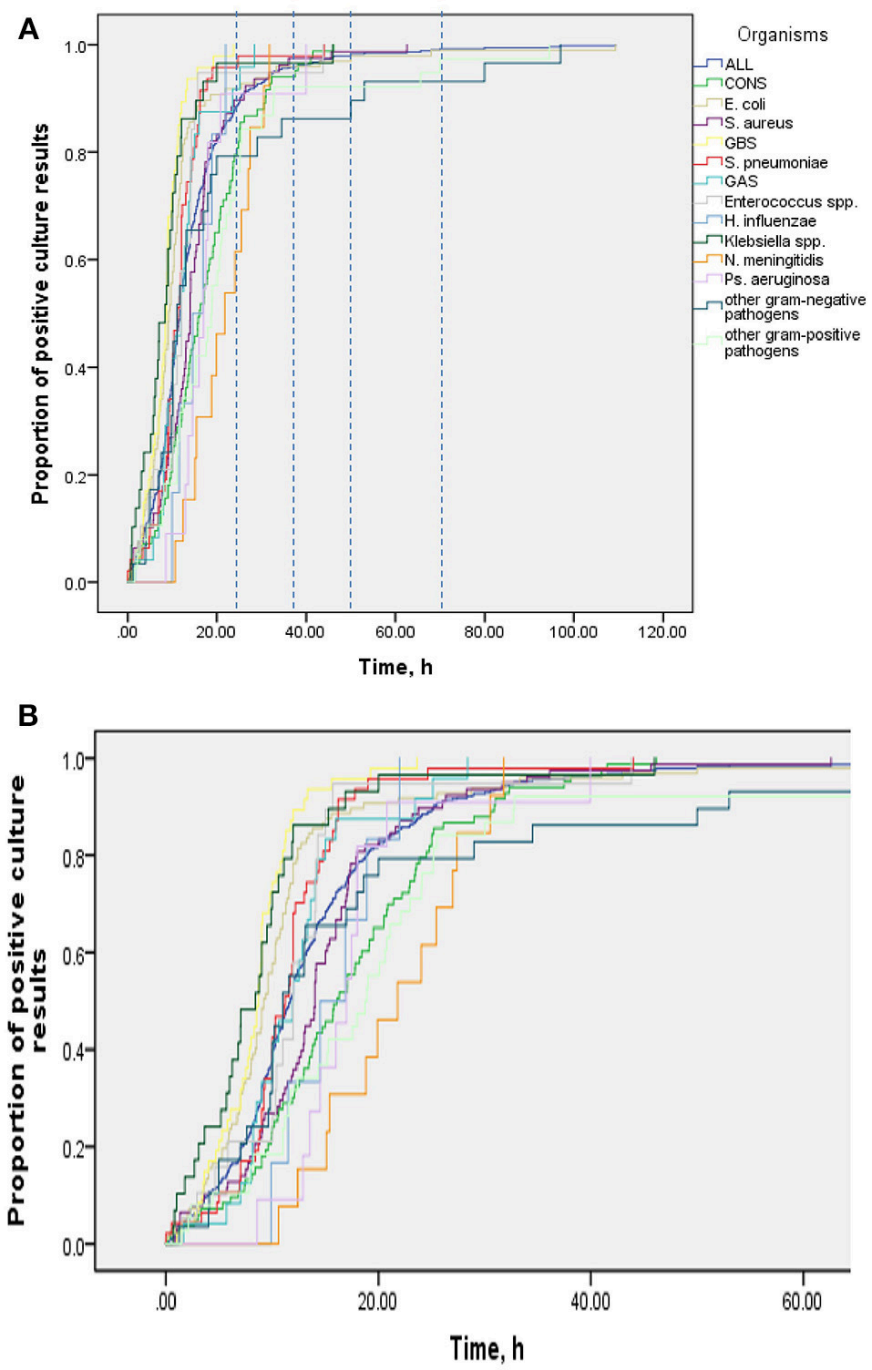
**(A)** TTP of all organisms, Kaplan-Meier-Curve. **(B)** cutout of **(A)**.

**Figure 2:**
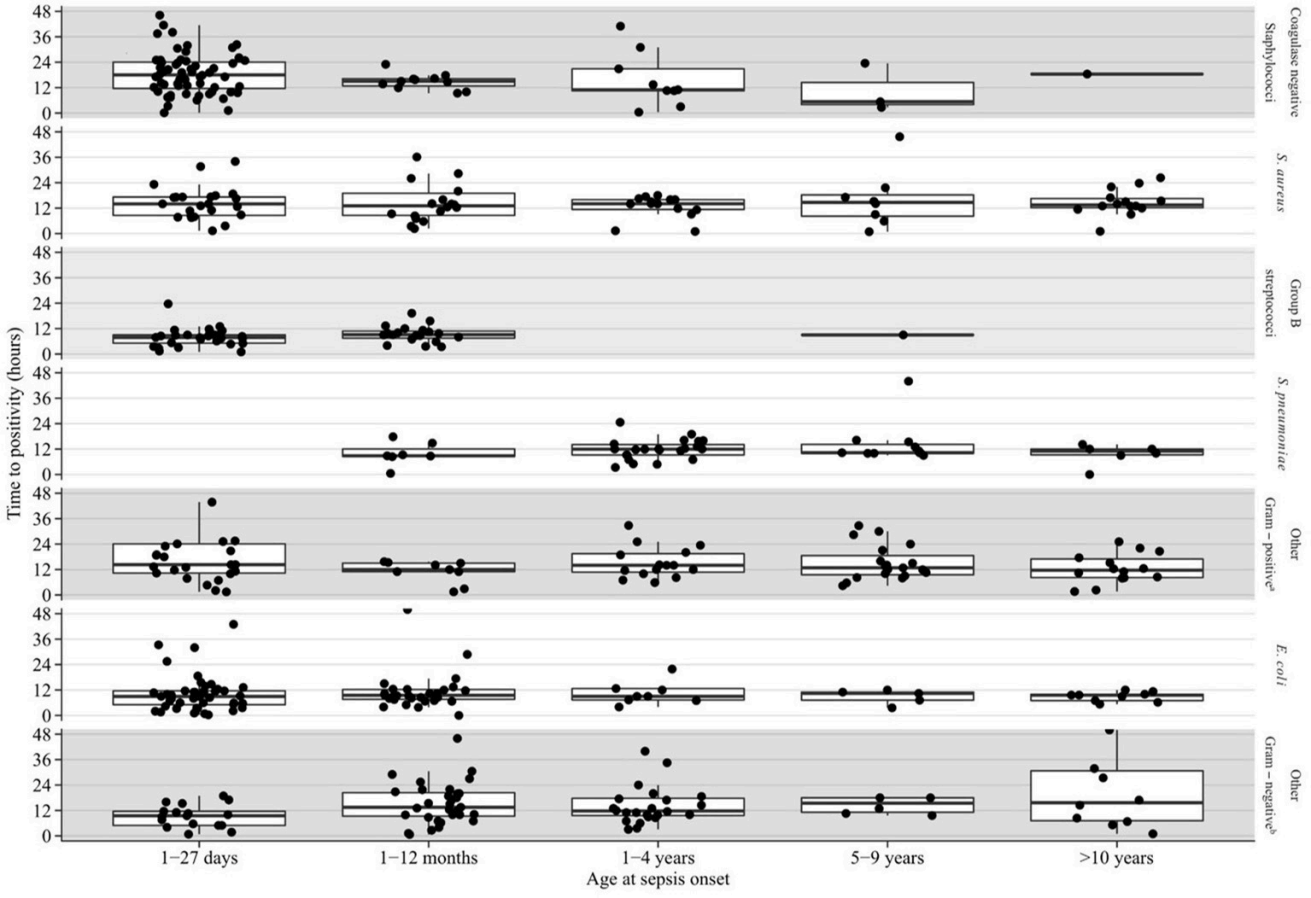
Blood culture TTP in selected bacterial pathogens by age group. ^a^Group A streptococcus, viridans group streptococci, other gram positive bacteria. ^b^Klebsiella species, *Neisseria meningitides, Pseudomonas aeruginosa*, other gram negative bacteria.

Table 2 shows results of multivariable analysis of TTP by patient and disease characteristics. TTP was independent of age, sex, presence of a comorbidity (see Table 3), site of infection, and severity of sepsis. Compared to coagulase-negative staphylococci, TTP was shorter in *Staphylococcus aureus* (−26%, 95% CI 5–43), group B streptococci (−56%, 95% CI 41–67), *Streptococcus pneumoniae* (−47%, 95% CI 26–63), and *Escherichia coli* (−44%, 95% CI 29–57). TTP of all organisms is demonstrated in Figures 1A,B and TTP of selected bacterial pathogens separated by different age groups in Figure 2.

**Table 2:** Median (IQR) TTP by patient and infection characteristics and adjusted^*^ estimates of TTP investigating potential predictive factors in a generalized linear model.

	**n (%)**	**TTP, Median (IQR), h**	**Multivariable model**	
			**Estimated TTP (95% CI)^a^**	**Adjusted *p*-value^b^**
Age groups				*p* = 1.0
<28 days	196 (38%)	11.3 (7.57–18.7)	Reference	
28–365 days	125 (24%)	11.0 (8.40–15.6)	0.95 (0.79–1.13)	
1–4 years	93 (18%)	12.0 (9.12–16.2)	1.01 (0.82–1.25)	
5–9 years	53 (10%)	11.9 (9.00–16.2)	0.97 (0.75–1.25)	
10–16 years	54 (10%)	12.0 (8.62–16.5)	0.94 (0.73–1.20)	
Sex				*p* = 0.3
Male sex	311 (60%)	11.3 (8.12–17.2)	Reference	
Female sex	210 (40%)	12.0 (8.50–16.8)	1.07 (0.94–1.22)	
Comorbidity				*p* = 0.1
No comorbidity	230 (44%)	12.0 (8.93–16.9)	Reference	
Comorbidity present	291 (56%)	11.0 (7.50–17.1)	0.85 (0.72–1.01)	
Severity of sepsis				*p* = 0.9
Sepsis	324 (62%)	11.7 (8.67–16.6)	Reference	
Severe sepsis	100 (19%)	12.4 (7.56–19.6)	0.96 (0.80–1.15)	
Septic shock	97 (19%)	11.3 (7.50–15.1)	1.00 (0.84–1.19)	
Site or type of infection				*p* = 0.1
Central-line associated bloostream	151 (29%)	11.9 (7.65–18.1)	Reference	
Primary bloodstream	104 (20%)	10.1 (7.17–14.4)	0.94 (0.76–1.17)	
Urinary tract	56 (11%)	11.0 (8.12–15.4)	1.27 (0.97–1.65)	
Pneumonia	45 (9%)	13.2 (10.3–16.2)	1.28 (0.95–1.74)	
Central nervous system	40 (8%)	10.0 (8.33–14.7)	0.94 (0.69–1.28)	
Gastrointestinal system	32 (6%)	11.8 (7.88–18.2)	1.32 (0.99–1.76)	
Bones and joints	30 (6%)	14.1 (11.3–20.1)	1.04 (0.72–1.49)	
Skin and soft tissue	30 (6%)	12.5 (9.35–16.7)	1.06 (0.79–1.42)	
Other specific infection type^b^	33 (6%)	12.0 (9.00–19.5)	1.20 (0.88–1.62)	
Pathogens				p < 0.001
Coagulase-negative staphylococci	84 (16%)	16.2 (10.4–23.1)	Reference	
*Staphylococcus aureus*	77 (15%)	14.0 (9.12–17.1)	0.74 (0.57–0.95)	
Group B streptococci	47 (9%)	8.70 (5.95–10.2)	0.44 (0.33–0.59)	
*Streptococcus pneumoniae*	46 (9%)	11.4 (9.05–13.9)	0.53 (0.37–0.74)	
Other Gram-positive bacteria^c^	82 (16%)	13.2 (10.0–19.8)	0.81 (0.63–1.03)	
*Escherichia coli*	97 (19%)	9.20 (6.68–12.0)	0.56 (0.43–0.71)	
Other Gram-negative bacteria^d^	88 (17%)	11.9 (8.54–18.7)	0.80 (0.62–1.04)	

* The adjusted model was adjusted for all variables listed

a Values and 95% CI are relative to the defined reference group

b *P-value from likelihood ratio test. Endocarditis, toxic shock syndrome, ear, nose, and throat infection, and other non-specified focal infections*.

c *Enterococcus spp., group A streptococcus, viridans group streptococci, other Gram positive bacteria*.

d *Haemophilus influenzae, Klebsiella spp., Neisseria meningitidis, Pseudomonas aeruginosa, other Gram negative bacteria*.

**Table 3:** Comorbidities detected in patients with 521 episodes of blood culture-proven sepsis.

	**Number (%) of episodes^c^**
Neurological or neuromuscular	17 (3)
Cardiovascular	35 (7)
Respiratory	30 (6)
Renal and urological	12 (2)
Gastrointestinal	37 (7)
Hematological or immunological	8 (2)
Metabolic	11 (2)
Other congenital or genetic defect	20 (4)
Malignant disease	46 (9)
Neonatal^a^	122 (23)
Surgery or burn	40 (8)
Technology dependence^b^	19 (4)
Solid organ transplantation	2 (<1)

a *Gestational age <27 weeks, or birthweight <750 g, or history of mechanical ventilation, or history of necrotising colitis*.

b *Central line or urinary catheter or ventriculoperitoneal shunt system present at sepsis onset, or total parental nutrition in a child that does not have any other comorbidity*.

c *More than one comorbidity might be present in a sepsis episode; therefore, numbers and percentages do not add up to 521 and 100, respectively*.

Of the 521 sepsis episodes, 100 (19%) were severe and 97 (19%) presented with septic shock. There was no significant association between 30-day in hospital mortality and TTP: in children who died in the first 30 days after sepsis onset, median TTP was 12.0 (IQR 7.50–16.3) compared to 11.7 (IQR 8.4–17.1) in those who survived (*p* = 0.8).

### Characteristics of sepsis episodes according to TTP thresholds (TTP >24 to ≤36 h, >36 to ≤48 h and >48 h)

By 24 h, 460 (88%) of 521 blood cultures were positive as were 498 (96%) and 510 (98%) by 36 and 48 h, respectively (Table 4). When comparing infants <90 days of age with those >90 days of age, proportions of positive blood cultures within 24, 36, and 48 h were almost identical with 88% vs. 89%, 96% vs. 95%, and 98% vs. 97%, respectively. Of the 11 blood cultures which became positive after 48 h, 4 were obtained in infants <90 days of age including 3 preterm babies with a gestational age of 25+6 weeks, 26+2 weeks and 28+4 weeks, representing 1.6% of all positive blood cultures in this age group. Three each grew *E. coli, Bifidobacterium longum* and *Fusobacterium* species, one grew *Staphylococcus aureus* and one grew *Capnocytophaga* species. Two of the 3 children with *E. coli* septicemia and a TTP of >48 h had urinary tract infections and no organ dysfunction. The third child, without any comorbidities, suffered from peritonitis with septic shock. Of the other 8 episodes with TTP >48 h, 4 patients presented with signs of septic shock. One of them, an extremely premature baby (gestational age 25+6), died. Similar to the overall proportion of patients with comorbidities (69%), 6 (55%) of the 11 patients with TTP >48 h had a comorbidity and 2 of them developed severe sepsis with septic shock.

**Table 4:** Patient characteristics with sepsis episodes by TTP thresholds.

		**TTP threshold n (%)**			
		**≤ 24 h**	**>24 - ≤36 h**	**>36 - ≤48 h**	**>48 h**
Overall n		460	38	12	11
Age (d)	<90	226 (49%)	20 (53%)	7 (58%)	4 (36%)
Prematurity	≤28+0	66 (14%)	9 (16%)	4 (33%)	2 (18%)
	28+1 −31+6	32 (7%)	5 (13%)	1 (8%)	1 (9%)
	>32+0 – 36+6	35 (8%)	1 (3%)	1 (8%)	–
HAI		199 (43%)	18 (47%)	8 (67%)	5 (45%)
Severity	Severe sepsis	82 (18%)	13 (34%)	5 (42%)	–
	Septic shock	87 (19%)	5 (13%)	1 (8%)	4 (36%)
Fatal outcome		32 (7%)	2 (5%)	2 (17%)	1 (9%)
Comorbidity		257 (56%)	19 (50%)	9 (75%)	6 (55%)
Focus present		295 (64%)	27 (71%)	12 (100%)	10 (91%)

## Discussion

We report on prospectively assessed TTP in a large national prospective cohort of children with blood culture proven sepsis. In our multivariate analysis, TTP was only dependent on pathogens, whereas sex, age, site of infection, presence of a comorbidity and severity of sepsis were not relevant.

Nine of 10 blood cultures turned positive within 24 h and 19 of 20 turned positive within 36 h after incubation. These findings contrast with the wide practice to treat children with suspected sepsis with antibiotics for >48 h while waiting for blood culture results. This “48 h rule” has historically evolved in the era prior to automated TTP recording systems. Our results indicate the need to critically reevaluate this rule.

Blood cultures represent one of the most widely used test in pediatrics, despite a low positivity rate, issues related to false positive tests due to contaminations, and false negative tests due to antibiotic pre-treatment and small inocula (16–18)., These limitations are increased if the cultures are obtained incorrectly, especially if the volume is inadequate (19). However, if positive, they confirm the diagnosis of bacteremia or sepsis and appropriate antibiotic treatment can be initiated (20). Fully automated blood culture systems with recording of TTP have evolved and several studies have since then been conducted to challenge the minimum of 48 h to observe or even treat a child with suspected bacteremia used by many centers (9, 10).

The recording of TTP in a large prospective population-based cohort of children with confirmed sepsis can inform clinicians in decision making about the appropriate length of empiric antibiotic treatment in the absence of a positive blood culture. Most clinicians stop antibiotic treatment if (a) the blood culture remains negative, (b) there is no focus of infection that would require continued antibiotic treatment, and (c) the clinical course of the disease is favorable. Yet, the chosen threshold of time after which blood cultures are highly unlikely to become positive will have a large influence on duration of antibiotic treatment. Longer periods of antibiotic treatment expose patients to risks such as medication errors, adverse events including selection of antibiotic resistance, nosocomial infections. Further, antibiotics increase the risk of necrotizing enterocolitis and death in preterm newborns (21). Moreover, prolonged hospital stays do increase health care costs and stress to patients, their relatives, and care-givers (22–24).

Our findings support previous studies performed in infants <90 days of age investigating TTP, which suggested that a shorter observation period and/or empiric antibiotic treatment, i.e., 24 or 36 h rather than the current practice of 48 h, might be appropriate (8–10). The decision by the treating physician to stop or continue antibiotics is not only based on blood culture result but also patient risk factors, clinical signs and severity of disease on presentation and during the course, response to treatment, inflammatory markers, and age. Procalcitonin-guided decision making has been shown to also guide the duration of antibiotic treatment in neonates with suspected early-onset sepsis (25, 26). It is well established that the likelihood to isolate an organism from blood culture increases with the amount of blood obtained for inoculation. Therefore, particularly in neonates blood cultures are often false negative (27). Unfortunately, there is limited data on the optimal volume in the pediatric age groups and different recommendations exist (28, 29).

In our study, the sensitivity of blood cultures increased only marginally (i.e., by 2%) when comparing a 48 h incubation threshold with a 36 h period. This demonstrates that the 48 h cut-off is arbitrary and based on tradition rather than strong evidence. However, the difficulty of decision making is exemplified by the fact that the longest TTP values in our study were 68 and 109 h for *E. coli* in the age groups 28–365 days and 1–5 years, respectively (Figure 2). Furthermore, our study was not designed to evaluate the prediction of which children, presenting with SIRS, will have a positive blood culture beyond 24 or 36 h. Hence, individual factors always need to be taken into account for decision making. Also, if the patient's clinical course is favorable and antibiotic treatment is stopped in the presence of a negative blood culture, parents still need to be advised to recognize signs and symptoms of concern and they need to be contactable for arrangement of a clinical reassessment as true pathogens might be detected after 36 or 48 h.

Not surprisingly, and also described in the literature, TTP varies by pathogen (30, 31). In our study, median TTP values were shortest for *E. coli* and GBS (approximately 9 h each) and longest for *S. aureus* and CoNS (14 and 16 h, respectively). While some studies found short TTP for organisms such as *Enterobacter*, E. coli or *S. aureus* correlated with poor outcomes in adults (30–32), median TTP value—irrespective of pathogen—did not predict poor outcome or admission and treatment on PICU in our study.

Also, a comparison of children <90 days of age and >90 days of age, as most studies in the literature investigated only children <90 days of age (9, 10), did not show any significant difference in their median TTP (11 h vs. 12 h). However, as infants <90 days of age and particularly neonates, represent a special pediatric cohort, especially in regards to their management, our data provides valuable information also on children >90 days of age. Children <90 days of age had significantly different sites of infection (e.g., primary blood stream infection) and sepsis episodes tend to be more severe. Also, as expected, pathogens differed significantly between these 2 groups, with *Escherichia coli* and CONS being the most common pathogens in infants <90 days of age and that is, in regards to *Escherichia coli* consistent with the literature (9, 10).

We did not see any significant differences in median TTP for children with or without comorbidity. A study in adults looking especially at patients with solid tumors or hematological disease found significantly lower TTPs in these patients compared with patients with benign tumors (33).

In our study, 42% of sepsis cases were hospital-acquired infections. Similar magnitudes have been reported before (34, 35). In a retrospective cohort study in South Africa investigating blood culture positive bacterial and fungal infections in children with a median age of 11.5 months, the proportion of hospital-acquired infections was 53.5% (34). Similarly, a study in South African neonates revealed a proportion of nosocomial infections of 62.2% of all positive blood cultures (35).

Whereas, as already mentioned above, most previous studies (retrospective or observational) investigated mainly neonates and infants <90 days of age or specific foci of infection (9, 10, 36), strengths of this study include the prospective, multicenter, population-based design and recruitment, use of a strict case definition, and inclusion of the entire pediatric age range with detailed capture of host and pathogen characteristics.

Our study had several limitations: Study centers that did not use automated laboratory systems had to be excluded. TTP “sensu strictu” (i.e., duration of incubation until blood culture becomes positive) must be distinguished from TTP “sensu latu” (i.e., time interval between collection of blood for culture and report of positive result to the treating physician). In our and most previously reported studies, TTP sensu strictu was analyzed. For patient management in the clinical context, TTP sensu latu reflects reality better than TTP sensu strictu and should be the subject of future studies. Also, the study protocol did not specify the maximum time allowed between obtaining blood cultures and processing them in the microbiology laboratory. Thus, bacteria may have started to replicate earlier in some episodes than formally measured, thereby leading to shorter TTP values. Furthermore, different blood culture systems were used in different study centers and the amount of inoculated blood, which is defined in each center according to the recommendations either given by the culture systems manufacturers or by local hospital guidelines, was not recorded. While these factors probably have increased heterogeneity of TTP findings in our study, they reflect a real world scenario in a pragmatic study design (37–39). Also, our study included children with comorbidities and it would have been interesting to carry out subgroup analyses on populations of special interest such as neutropenic patients. However, as numbers of specific comorbidities were low, such analyses would not have been meaningful. Finally, our study was based on children with positive blood cultures, and we cannot comment on blood culture negative bacterial infections. In addition, the study was not designed to analyse the performance of other clinical and laboratory criteria to guide antimicrobial therapy. As per international best practice, infectious diseases and/or antimicrobial stewardship team advice was routinely sought in the study centers once blood culture positivity was known.

In conclusion, blood cultures were positive within 36 h of incubation in 90% of children with sepsis. TTP was <24 h in the great majority of children with sepsis, but occasionally it was >36–48 h in individual sepsis episodes. Therefore, a strict and general rule to treat or observe all children for at least 48 h is not justified; rather, the decision to continue with empiric antibiotic treatment in the absence of a positive blood culture should be reconsidered after 24 and 36 h and antibiotic treatment should be stopped if the diagnosis of sepsis cannot be held up. Future studies are needed to test whether empiric antibiotic treatment in children with negative blood cultures can be safely reduced to <48 h and to identify factors that would allow to predict which children with SIRS will still have positive blood cultures beyond 36 h of incubation. Still, TTP is an important pillar on which the decisions for or against antibiotic treatment and its duration should be based. Therefore, standardized measurement of TTP in children with sepsis continues to be valuable, and ongoing analyses should be encouraged.

## Author contributions

ADi, CB, EG, MS, LS and UH: conception or design of the work. ADi, CB, EG, MS, SB-S, and UH: data collection. ADi, CB, PA, LS, and UH: data analysis and interpretation. ADi: drafting the article. CB, SB-S, EG, MS, PA, LS, and UH: critical revision of the article. Final approval of the version to be published and agreement to be accountable for all aspects of the work in ensuring that questions related to the accuracy or integrity of any part of the work are appropriately investigated and resolved: all authors.

### Conflict of interest statement

The authors declare that the research was conducted in the absence of any commercial or financial relationships that could be construed as a potential conflict of interest.
