# Supplementary material for: Time-to-Positivity of Blood Cultures in Children With Sepsis
Source: Front Pediatr. 2018 Aug 8;6:222. doi: 10.3389/fped.2018.00222 (PMC6092514; doi:10.3389/fped.2018.00222)
Supplement: Supplementary file 1 [file Table_1.DOCX]

**Supplement**

Table 1: Comparison of selected variables in children <90 days compared to older children.*

|  | Episodes in children <90d old | Episodes in children ≥90d old | Multivariable model p Value^a^ |
| --- | --- | --- | --- |
| Time to positivity (median (IQR)), h | 11.0 (7.50-17.2) | 12.0 (9.00-16.9) | p = 0.5 |
| Sex |  |  | p = 0.3 |
| Male sex | 153 (60%) | 158 (60%) |  |
| Female sex | 104 (40%) | 106 (40%) |  |
| Comorbidity |  |  | p = 0.6 |
| No comorbidity | 99 (39%) | 131 (50%) |  |
| Comorbidity present | 158 (61%) | 133 (50%) |  |
| Severity of sepsis |  |  | p < 0.001 |
| Sepsis | 126 (49%) | 198 (75%) |  |
| Severe sepsis | 88 (34%) | 12 (5%) |  |
| Septic shock | 43 (17%) | 54 (20%) |  |
| Site or type of infection |  |  | p < 0.001 |
| Central-line associated bloodstream | 86 (33%) | 65 (25%) |  |
| Primary bloodstream | 82 (32%) | 22 (8%) |  |
| Urinary tract | 30 (12%) | 26 (10%) |  |
| Pneumonia | 14 (5%) | 31 (12%) |  |
| Central nervous system | 14 (5%) | 26 (10%) |  |
| Gastrointestinal system | 15 (6%) | 17 (6%) |  |
| Bones and joints | 2 (1%) | 28 (11%) |  |
| Skin and soft tissue | 8 (3%) | 22 (8%) |  |
| Other specific infection type^b^ | 6 (2%) | 27 (10%) |  |
| Pathogens |  |  | p < 0.001 |
| Coagulase-negative staphylococci | 66 (26%) | 18 (7%) |  |
| *Staphylococcus aureus* | 30 (12%) | 47 (18%) |  |
| Group B streptococci | 42 (16%) | 5 (2%) |  |
| *Streptococcus pneumoniae* | 0 (<1%) | 46 (17%) |  |
| Other gram-positive bacteria^c^ | 31 (12%) | 51 (19%) |  |
| *Escherichia coli* | 61 (24%) | 36 (14%) |  |
| Other gram-negative bacteria^d^ | 27 (11%) | 61 (23%) |  |

*Data are numbers (%) unless indicated otherwise. The adjusted model was ajdusted for all variables listed ^a^P value from likelihood ratio test. ^b^Endocarditis, toxic shock syndrome, ear, nose, and throat infection, and other non-specified focal infections. ^c^*Enterococcus spp*, group A streptococcus, viridans group streptococci, other Gram positive bacteria. ^d^*Haemophilus influenzae*, *Klebsiella spp*, *Neisseria meningitidis*, *Pseudomonas aeruginosa*, other Gram negative bacteria
