# Supplementary material for: Time-to-Positivity of Blood Cultures in Children With Sepsis
Source: Front Pediatr. 2018 Aug 8;6:222. doi: 10.3389/fped.2018.00222 (PMC6092514; doi:10.3389/fped.2018.00222)
Supplement: Supplementary file 2 [file Table_2.DOCX]

**Supplement**

Table 2: TTP (in hours) of 521 bacterial pathogens isolated from blood cultures by sex and study center (A-F)

| TTP |  | A | B | C | D | E | F | Total |
| --- | --- | --- | --- | --- | --- | --- | --- | --- |
| Range | All | 4 - 97 | 1 - 66 | 0 - 109 | 7 - 33 | 0 - 63 | 1 - 33 | 0 - 109 |
|  | Female | 4 - 68 | 1 - 66 | 0 - 94 | 7 - 28 | 1 - 50 | 6 - 33 | 0 - 94 |
|  | Male | 4 - 97 | 2 - 43 | 0 - 109 | 7 - 33 | 0 - 63 | 0 - 24 | 0 - 109 |
| Median | All | 11 | 10 | 10 | 14 | 12 | 11 | 12 |
|  | Female | 12 | 10 | 11 | 13 | 12 | 11 | 12 |
|  | Male | 11 | 11 | 9 | 14 | 12 | 10 | 11 |
| IQR | All | 9-15 | 8-23 | 8-17 | 11-17 | 8-17 | 7-16 | 8-17 |
|  | Female | 9-14 | 8-21 | 6-20 | 12-16 | 8-16 | 9-15 | 8-17 |
|  | Male | 9-15 | 8-24 | 4-18 | 11-17 | 10-22 | 5-16 | 8-17 |
